# 106. The risk, timing and clinical impact of postnatal cytomegalovirus transmission on preterm infants born less than 29 weeks of gestational age: A prospective multicenter cohort study

**DOI:** 10.1093/ofid/ofaf695.041

**Published:** 2026-01-11

**Authors:** Y U ichiro Sugiyama, Ryuichi Tanaka, Michio Suzuki, Tetsuo Koshizuka, Keita Takahashi, Juri Koizumi, Takako Suzuki, Yoshiaki Sato, Yoshinori Ito, Yuka Torii

**Affiliations:** Japanese Red Cross Aichi Medical Center Nagoya Daiichi Hospital, Nagoya, Aichi, Japan; Nagoya University Hospital, Nagoya, Aichi, Japan; Anjo Kosei Hospital, Anjo, Aichi, Japan; Gifu Pharmaceutical University, Gifu, Gifu, Japan; Gifu Pharmaceutical University, Gifu, Gifu, Japan; Gifu Pharmaceutical University, Gifu, Gifu, Japan; Nagoya University Graduate School of Medicine, Nagoya, Aichi, Japan; Nagoya University Hospital, Nagoya, Aichi, Japan; Aichi Medical University, Naogya, Aichi, Japan; Nagoya University Graduate School of Medicine, Nagoya, Aichi, Japan

## Abstract

**Background:**

As the survival rate of extremely preterm infants has increased, postnatal CMV infection (pCMV) has been considered a potential contributor to morbidities in this population. This study aimed to clarify the risk, symptoms, and complications of pCMV.

**Methods:**

This is a multicenter prospective cohort study of infants born < 29 weeks of postmenstrual age (PMA) with CMV-PCR negative results in urine tests within 2 weeks of birth. Serial viral loads in breast milk and infant blood and urine were monitored every 2 weeks until 36 weeks of PMA. Binding, neutralizing and non-neutralizing functions of maternal serum CMV-specific antibodies, including antibody-dependent cellular cytotoxicity (ADCC) and antibody-dependent cellular phagocytosis (ADCP), were assessed for the infection risk.

**Results:**

During the study period from Dec 2021 to Sep 2024, 139 infants were enrolled. Eighty-three (59%) of these infants were born to CMV-seropositive mothers. The infants' gestational age and birth body weight were 26 weeks (IQR 24-27), and 763 g (515-1011), respectively. The prevalence of pCMV by 36 weeks of PMA was 14/139 (10%) overall and 13/83 (16%) in infants born to seropositive mothers.

Symptoms associated with pCMV were transient neutropenia (< 1000/µL) (pCMV+ 42% vs. pCMV- 11%, p< 0.01) and acute respiratory exacerbation after 3 weeks of age (31 vs. 11%, p=0.08).

Infants with pCMV were born at a lower gestational age (24 vs 25 weeks, p=0.06) and experienced premature rupture of membranes more than 24 hours before birth (58% vs 17%, p=0.01). pCMV was associated with a higher CMV load in maternal milk at 2 weeks (141820 vs 7815 copies/µL, p< 0.01) and 4 weeks (40296 vs 8415 copies/µL, p< 0.04) after birth. In maternal serum, CMV-specific antibody levels were not different, but ADCP activity was lower (85% vs 78%, p=0.01) in the pCMV group. In addition, total serum IgG levels tended to be lower in the pCMV group (245 vs. 340 mg/dL, p=0.07).

**Conclusion:**

Approximately 16% of extremely preterm infants born to CMV-seropositive mothers develop pCMV by term equivalent age. Risk factors for infection include low maternal ADCP activity, lower gestational age, and premature rupture of membranes more than 24 hours prior to birth. Neutropenia and chronic lung disease may be symptoms of pCMV.

**Disclosures:**

All Authors: No reported disclosures

